# Combined assessment of tuberculosis case notification rate and infection control at health facilities of Dale districts, Sidama Zone, Southern Ethiopia

**DOI:** 10.1371/journal.pone.0242446

**Published:** 2021-10-12

**Authors:** Makka Adam Ali, Ermias Sissay Balcha, Adugna Abdi Woldesemayat, Lopisso Dessalegn Tirore

**Affiliations:** 1 Department of Oncology, Faculty of Medicine, Lund University, Lund, Sweden; 2 Department of Cellular and Molecular Biology, Faculty of Sciences, Addis Ababa University Addis Ababa, Ethiopia; 3 Department of Microbiology, Faculty of Medicine, Yirgalem Hospital Medical College, Yirgalem, Ethiopia; 4 School of Medical Laboratory Science, College of Health Sciences, Hawassa University, Hawassa, Ethiopia; 5 Department of Biotechnology, College of Biological and Chemical Engineering, Addis Ababa Science and Technology University, Addis Ababa, Ethiopia; Indian Institute of Technology Delhi, INDIA

## Abstract

**Background:**

Mycobacterium tuberculosis (TB) is the deadliest disease that claims millions of deaths globally. Ethiopia is among the countries heavily hit by the disaster. Despite the effective directly observed treatment and TB infection control (TBIC) measures provided by the world health organization (WHO), the rate of new cases increased daily throughout the country. Healthcare workers (HCWs) are at highest risk serving without having the necessary facility in place while overcrowding of patients exacerbated TB transmission. The study aimed to assess TBIC implementation and analyze case notification rate (CNR) of smear-positive pulmonary TB in the selected health facilities at Dale district, Sidama Zone, Southern Ethiopia.

**Methods:**

Seven health care facilities have been visited in the study area and smear-positive pulmonary TB notification rate was determined retrospectively during the years 2012 to 2014. Data on smear positive test results and demographic characteristics were collected from the TB unit registries. A structured questionnaire, facility survey, and observation checklists were used to assess the presence of TBIC plans at the health care facilities.

**Results:**

The overall case notification rate of smear-positive pulmonary tuberculosis was 5.3% among all 7696 TB suspected patients. The odds of being diagnosed with smear-positive TB were 24% more in males than in females (adj OR = 1.24, 95% CI: (1.22, 1.55). Moreover, in the study area, only 28% of the facilities have been practiced TB infection control and 71% of the facilities assigned a focal person for the TBIC plan. The implementation of environmental control measures in the facilities was ranged between 16–83%. N95 particulate respirators were found only in 14% of the facilities.

**Conclusion:**

TB CNR in Dale district was low. Moreover, implementations of TBIC in Dale district health facilities were poor when the survey was done. Hence, urgent measures should be taken to reverse the burden of TB.

## Introduction

Tuberculosis (TB) has affected mankind since antiquity and has been found in ancient Babylonian and Chinese writings [1, 2]. TB is a deadliest chronic, infectious disease caused by Mycobacterium tuberculosis [3]. According to the WHO report, 9.27 million new active cases have occurred annually which corresponds to an incidence of 139 per 100,000 populations throughout the world [4]. Asia is the highest hit (55%) by TB cases followed by Africa (31%). The highest incidence rate (363 per 100,000 populations) was recorded for Africa which was possibly due to the highest rate of HIV infection [5, 6].

Ethiopia is among the countries heavily affected by tuberculosis [5]. Ethiopia has been classified by the WHO as the 7^th^ among the 22 significantly burden countries with TB infection and again has been ranked 3^rd^ in terms of the number of extra-pulmonary TB cases globally [7]. The annual TB incidence and mortality rate is estimated to be 341/100,000 and 73/100,000 respectively [8]. A well-organized TB control program was started in Ethiopia in 1992 and scaled up to over 71% of the geographic coverage [7, 8]. Now, ninety-five percent of the health institutions give Directly Observed Treatment (DOT) service for the communities [8]. Unfortunately, 40% of them don’t have an access to health service and the treatment success rate is about 76% [9]. It is noted that, the aggravation of TB for many years have largely been attributed to the failure to carry out rapid CNR and infection control measures [2, 10]. Case notification is an important element of the DOT strategy and is influenced by individual (care-seeking behavior), social (access to health care), and biomedical (diagnostic capability) factors [10]. In the developing world, many people with TB live and die without the disease ever being diagnosed or face delay experiencing unprecedented challenges in diagnosis and treatment [11]. Studies from sub-Saharan Africa have reported 50 to 180 days delays in TB case notification [12]. In Ethiopia, despite an accelerated decentralization of DOT, the CNR of smear-positive TB in most TB programs is less than the global target of 70% [13–15].

However, TB case detection and treatment outcomes vary between regions in Ethiopia regardless of the residential places [16]. This ultimately results in either over or underestimation of CNR to the true population for the defined administrative areas [17]. In most developing countries, infectious (smear-positive) TB patients are managed at health-care facilities, without effective infection control measures in place. It was shown that such a situation is worsened in a condition where there is overcrowding of patients and lack of appropriate infrastructure which might leads to a delayed diagnosis and treatment [18, 19]. In response to such yearly round pandemic, Centers for Disease Control and Prevention (CDC) and the WHO have already developed and published TBIC plan or guidelines based on a three-level hierarchy of controls: administrative practice control, environmental control and respiratory protection control [20, 21].

Although numerous studies have been undertaken about the prevalence and incidence of tuberculosis across couple of regional states of Ethiopia, no combined studies have been conducted on tuberculosis CNR and Infection control measures at health facilities level particularly in Dale districts [22]. Hence, this is the only pioneering study motivated to fill these gaps; it aims to determine the case notification rate of TB and describes tuberculosis infection control measures implemented by Dale district health facilities, Sidama Zone, Southern Ethiopia.

## Methods

### Study area

The study was carried out in the southeast part of Ethiopia, Sidama zone, Dale district. Dale district is one of the districts in Sidama zone located in the Great Rift Valley at 6°39’ to 6°50’N and 38°18’ to 38°31’E. The district covers a total area of about 30,212 hectare which comprises of 36 kebeles (the smallest administrative units next to district in Ethiopia). Health centers of Mesenkela, Semen Kege, Boa Badagelo, BeraTolicha, Dagiya, Megera and Mato were among the kebeles in the Dale district that the study focused on. Based on Central Statistics Authority Census data of the 2007, Dale district has a total population of about 228,638, of which 12.51% are urban dwellers [23].

### TB diagnosis

We used the National Tuberculosis, Leprosy and TB/HIV Prevention and Control Programme of Ethiopia for the diagnosis of TB cases and for case definitions [24]. The health care facilities identify suspects, carried out sputum microscopy, and refer patients to hospitals when cases are smear-negative and extra pulmonary.

### Case definition

Extra pulmonary TB (EPTB) is tuberculosis within an extra pulmonary site, lymph nodes, meninges, skin, abdomen, pleura, genitourinary tract, joints and bones, in the body other than the lungs. The diagnosis is conducted using fine needle aspiration (FNA) for histopathological evidence from a biopsy, or biochemical analysis of fluids from extra pulmonary site, which is based on clinician’s decision. However, a patient with three consecutive sputum smear negative for Acid-Fast Bacillus (AFB) at health care facilities and with no clinical response for a full course of broad spectrum antibiotic therapy for two weeks, while suspected of EPTB at health care facilities were referred to hospital for further radiological and histopathological diagnosis. The study does not reflect information on extra-pulmonary TB cases as no smear test results were found in the record books.

Pulmonary TB smear-positive (PTB+) diagnosis is based on the presence of at least two positive initial sputum smears for AFB by direct microscopy or at least one smear positive for AFB and with chest radiographic abnormalities consistent with active pulmonary TB determined by clinician’s decision.

Pulmonary TB smear-negative (PTB-) case is based on the diagnosis of at least three initial sputum smear examinations negative for AFB by direct microscopy although presented with symptoms of TB, repeat smear negative with chest radiographic abnormalities consistent with active pulmonary TB, no response to broad spectrum antibiotics for two weeks, as well as decision determined by clinician’s.

### Study design and data collection

A quantitative-based retrospective design was adapted to study the TB case notification rates registered from years between January 1, 2012 and December 31, 2014 consecutively. The case notification rates on the study area were conducted using a record review of smear test results. Socio-demographic information such as age, sex was also collected from the record books. TB infection control plan was also assessed at each health facility of Dale district using the guidelines of CDC and WHO [20]. The survey was assessed through interviews of the facility managers or officer in charge of the TB clinic and by observation of TBIC practices. The survey questionnaire (S1 Appendix) contains information such as the number of staff by calendar year, whether the facility has a fast track of examination for TB suspects or patients, availability of ventilated waiting area designated specifically for the treatment of TB patients load etc. Questions such as the availability of TB infection control measures were also included based on the recommended guidelines (WHO, 2019) for the prevention of TB in health care facilities [21]. TB infection control measures were taken to be TBIC plan, HCWs training, prompt identification of TB suspects, triage, patient education about TB, sputum collection practices (in a well-ventilated area), well-ventilated facilities, and use of protective wear [21].

### Data analysis

Descriptive statistics was used to assess the implementation of TBIC measures and determination of case notification rate using SPSS version 16.0. Simple frequency tables and charts were used to compare TBIC plan tools and their implementation in the health care facilities. The same statistical data were also collected for case notification rate of smear-positive TB cases for over 3 consecutive years (2012–2014). Having obtained a year based population data for the study area, case notification rates were computed using the population for different years as a denominator and notified cases as a numerator [16]. Proportions of smear- positivity were also analyzed based on age groups, gender, and fiscal year. TB infection control assessment was made based on a simple scoring system: tally on the presence or absence of systems, documents or committees, whether specific control measures were implemented, or not. Proportions of compliance were determined from the scored survey. The denominator for every proportion corresponding to a question was represented by the number of facilities. A comparison was made based on the proportions scored by facilities. P-value < 0.05 was considered statistically significant.

### Ethical approval

Ethical clearance was obtained from the scientific and ethical review committee of Yirgalem hospital medical college and regional health bureau of Southern Ethiopia. In the survey, study objectives, goals, and procedures were explained to the participant verbally and informed written consent was obtained. Moreover, participants were informed that their participation in the survey is completely voluntary and that they could decline not to participate at any time given if they want to do so even without giving reason for withdrawal. Prior to analysis, all medical records were kept in a secure place to help maintain the confidentiality of clinical information of all existing cases.

## Results

### Patient turnover

**Fig 1:**
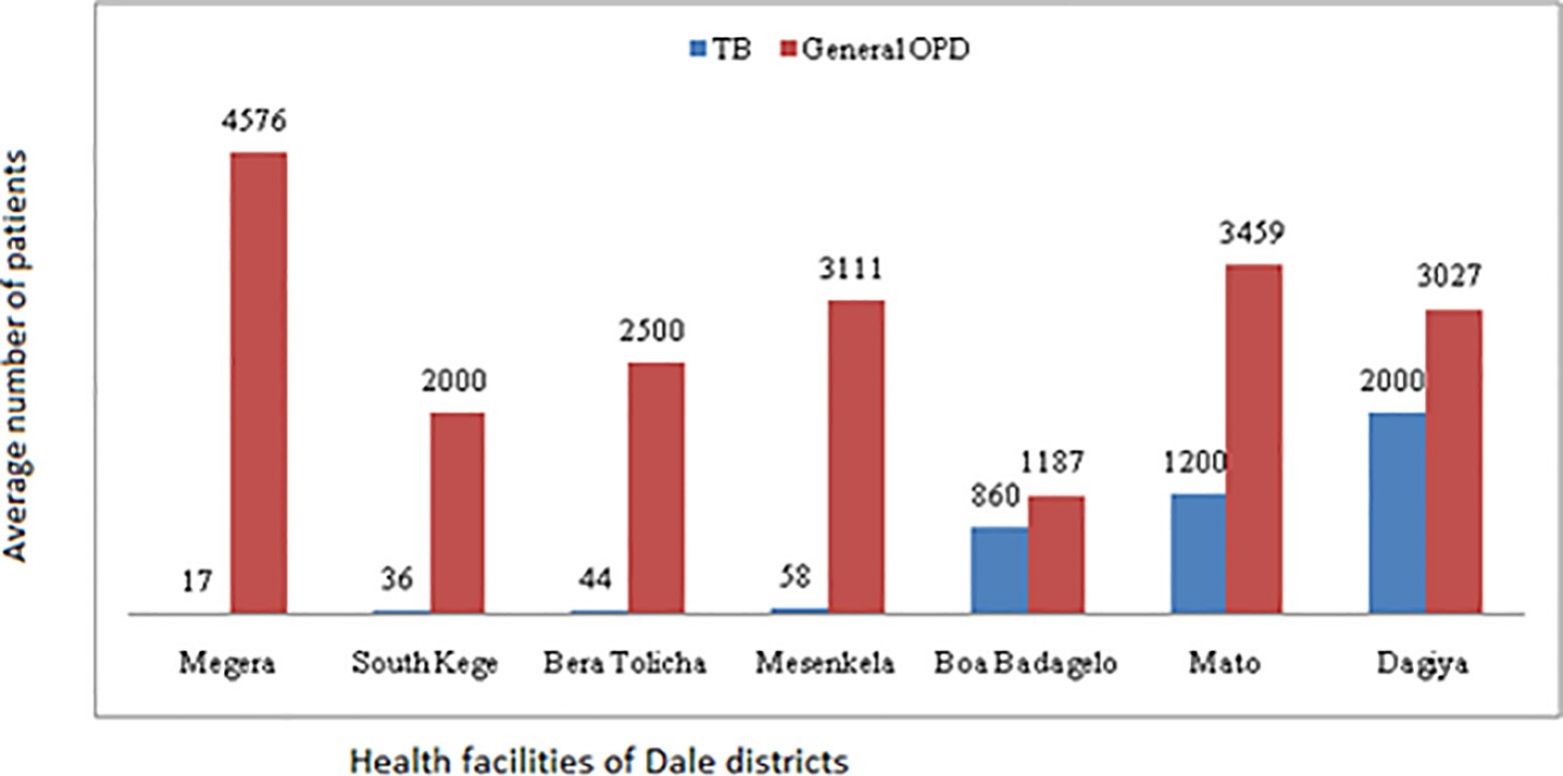
Total OPD patients and TB suspected patients per year in the Dale district health facilities, Sidama Zone, Southern Ethiopia.

The study was conducted in a government-owned seven health centers. All the facilities had a general outpatient department (OPD) and TB clinic. The average annual number of patients turns over in the general OPD was 3027 with interquartile range (IQR) between 2000 and 3459 patients. The average number of TB suspected patients per year was 58 with IQR of 36 to 1200 patients per year. Though services for TB diagnosis and treatment existed in all studied facilities, the number of TB patients’ turnover was found to vary across the facilities. The number of patients in all studied facilities is shown in Fig 1.

### TB infection control measures in the facilities

#### Availability of managerial measures

**Fig 2:**
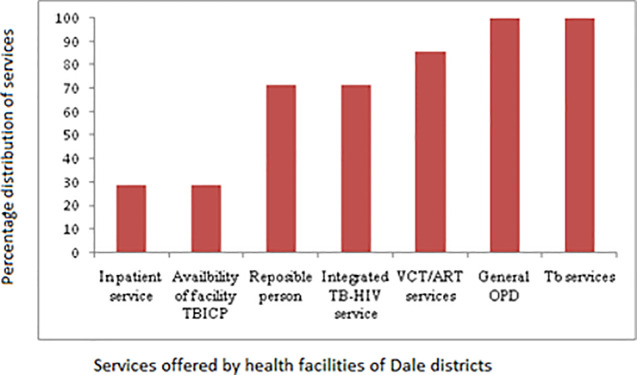
Distribution of TB related services other than TBIC plan and in-patient service in the Dale district health facilities, Sidama Zone, Southern Ethiopia.

It was found that only 2/7 (28.6%) of the facilities had a TB infection control plan and similarly in-patient service. TB clinic focal persons were present in 5/7 (71.4%) of the facilities. The distribution of other services in the facilities is displayed in Fig 2.

#### Administrative measures in the facilities

TBIC plan has several elements that are considered very instrumental for effective control of TB infection. Health facilities were asked or observed for the presence or absence of administrative TBIC measures. Out of the seven health facilities, only one (14.3%, n = 1/7) witnessed participating in operational research.

**Fig 3:**
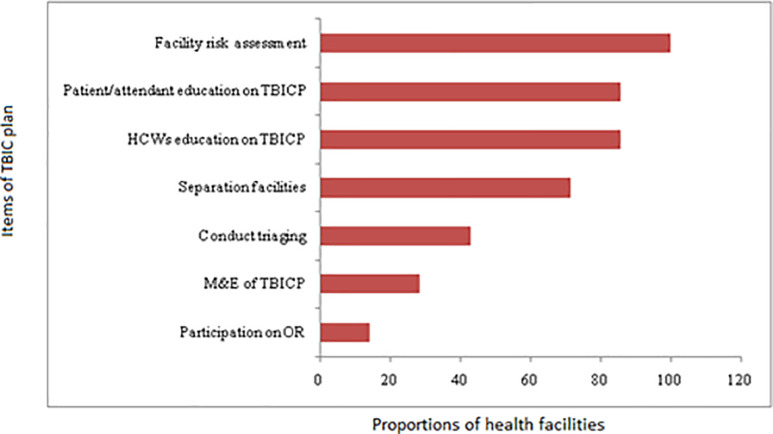
The proportion of health facilities with various items of TBIC plan in the Dale district, Sidama Zone, Southern Ethiopia.

Monitoring and evaluation of these activities are also an integral part of the TBIC program. The current study revealed that only 2 of the investigated facilities (28.6%) had active TBIC monitoring and evaluation performance. No facility was observed or reported to practice facility risk assessment for taking appropriate measures when and where necessary. Only 42% of the facilities conducted triaging and separating of TB suspected patients from others. Proportion of facilities with other TBIC activities is shown in Fig 3.

#### Clinic organization and presence of other administrative measures

In the studied facilities, it was found that all of the facilities had a separate HIV clinic and conducted screening of TB infection for people with HIV. However, only 71.4% of the facilities had the TB screening checklist and no facility had a separate waiting area for HIV patients. All facilities provide health education for adherence to TB treatment patients. Furthermore, 5/7 (71.4%) of the facilities educated patients on cough hygiene, but only 3/7 (42.8%) of the facilities displayed educational posters, Table 1.

**Table 1:** The facilities’ clinic organization and presence of other clinical services in Dale district, Sidama Zone, Southern Ethiopia.

Variable	No.	%
Screening TB among HIV patients	7	100
Separate HIV clinic	7	100
Ensuring adherence to prescription	7	100
Cough etiquette	5	71.4
Checklist to aid screening	5	71.4
Poster on cough	3	42.9
Package of prevention (HIV,TB) for HCWs	0	
Are staff members screened for TB?	0	
Presence of separate sites for HIV care & treatment	0	

### Environmental and personal protective measures

**Fig 4:**
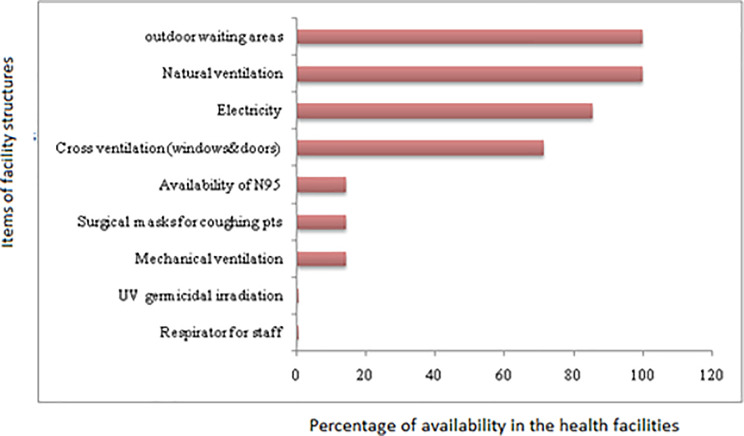
Availability of facility structures for appropriate TBICP measures in Dale district, Sidama Zone, Southern Ethiopia.

It was found that none of the facilities had UV germicidal irradiation and only 14.3% of them were observed to have N95 respirator for their staff. Most Health care workers were observed wearing surgical masks to protect themselves. Other infrastructures that don’t require high tech facilities such as natural ventilation, outdoor waiting areas, and electricity were present in almost all of the facilities. Moreover, 71% of the facilities have cross natural ventilation (window to door) and only 1/7 (14.3%) of the facilities has mechanical ventilation, Fig 4.

### Level of implementing the pillar’s of TBIC plan

**Fig 5:**
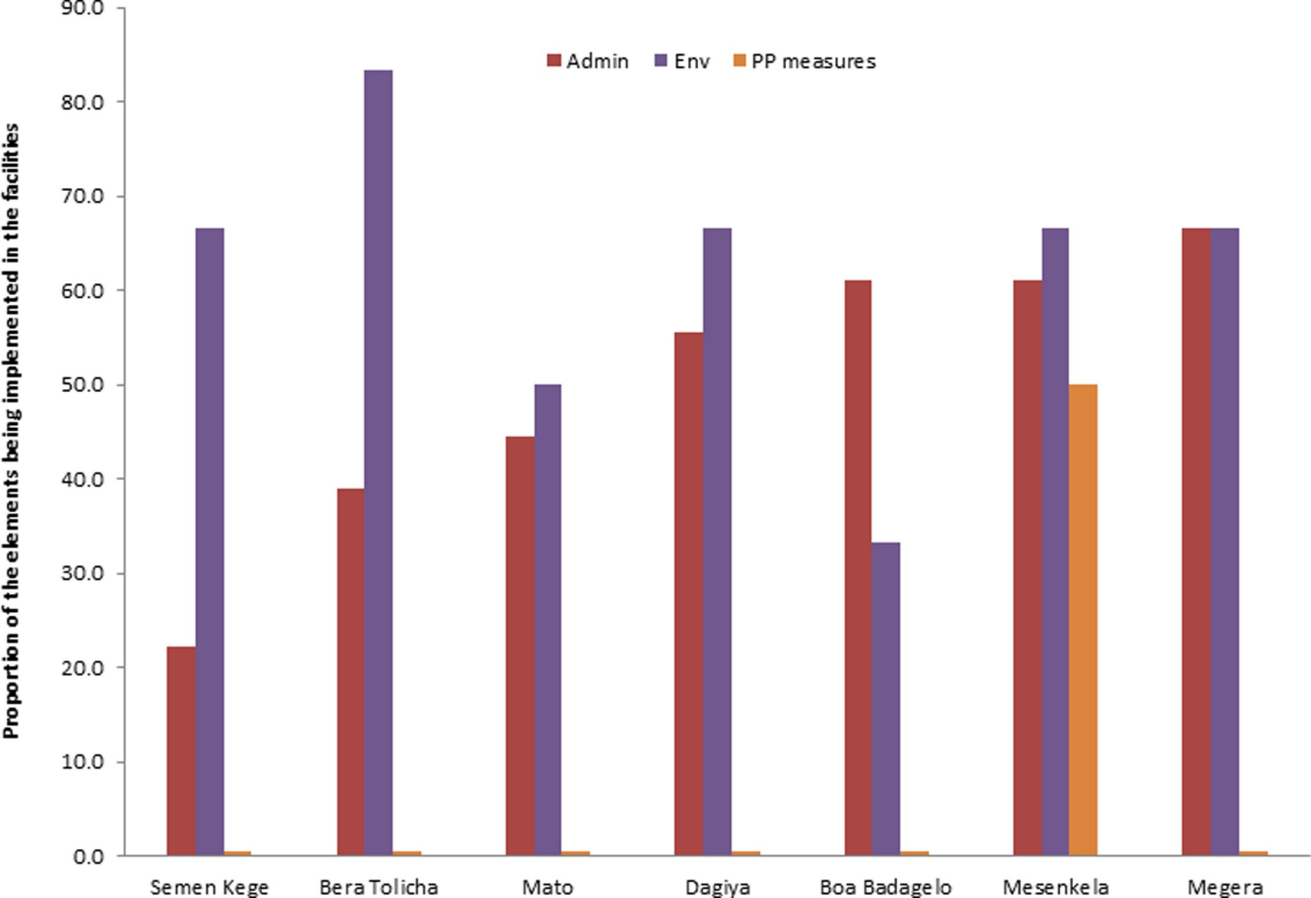
The three hierarchies of TB infection control program for each studied health facilities in Dale district, Sidama Zone, Southern Ethiopia.

The study also tried to assess the extent to which the three levels TBIC hierarchy, namely, administrative, environmental, and personal protection controls were being implemented in the facilities. It was observed that almost all facilities lack any of the items for personal protection controls except Mesenkela health center (Fig 5). BeraTolicha health center found to have a maximum number of environmental control measures while Megera health center had the highest number of administrative controls, Fig 5. The total score range of the audit tool was found to be between -14 and 7 with a mean value of -2.

### Case notification rates of smear-positive TB

#### TB incidence rate

A total of 7696 TB cases were found from each of the health centers’ attendance record books from 2012 to 2014 Dale district, Sidama Zone. The female to male ratio was 147:100. The median age of the patients was 35.9 (SD = 14.7). A total of 406 TB smear-positive patients were also found among 7696 TB suspects, Table 2.

**Table 2:** Background characteristics of the study subjects and the catchment size of the facilities, Dale district, Sidama Zone, Southern Ethiopia.

Variable	* *	2012(%)	2013(%)	2014(%)	Total(%)
**Sex**	Male	745 (38.4)	1231 (39.5)	1136 (43.0)	3112 (40.4)
	Female	1197 (61.6)	1873 (60.1)	1489 (56.4)	4559 (59.3)
	Missing	0 (0.0)	11 (0.4)	14 (0.5)	25 (0.3)
**Age**	0–5	4 (0.2)	3 (0.1)	5 (0.2)	12 (0.2)
** **	6–14 years	141 (7.3)	171 (5.5)	142 (5.4)	454 (5.9)
** **	15–24	295 (15.2)	394 (12.6)	312 (11.8)	1001 (13.0)
** **	25–34	405 (20.9)	817 (26.2)	695 (26.3)	1917 (24.9)
** **	35–50	687 (35.4)	1104 (35.4)	1144 (43.3)	2935 (38.1)
** **	>50	4 (0.2)	18 (0.6)	9 (0.3)	31 (0.4)
** **	Missing	406 (20.9)	608 (19.5)	332 (12.6)	1346 (17.5)
** **	Mean, SD	36, 15.9	36.2, 14.8	35.6, 13.5	35.9, 14.7
**Catchment population**	Boa Badagelo	31837	32653	33559	NA
	BeraTolicha	27684	28393	29110	NA
	Mesenkela	26210	26882	27627	NA
	Dagiya	25912	26576	27313	NA
	Semen Kege	20257	20776	21352	NA
	Mato	43177	44284	45512	NA
	Megera	18807	19289	20374	NA
	Total	193884	198853	204847	NA

NA = not available.

### Percent proportion of smear-positive TB cases

**Fig 6:**
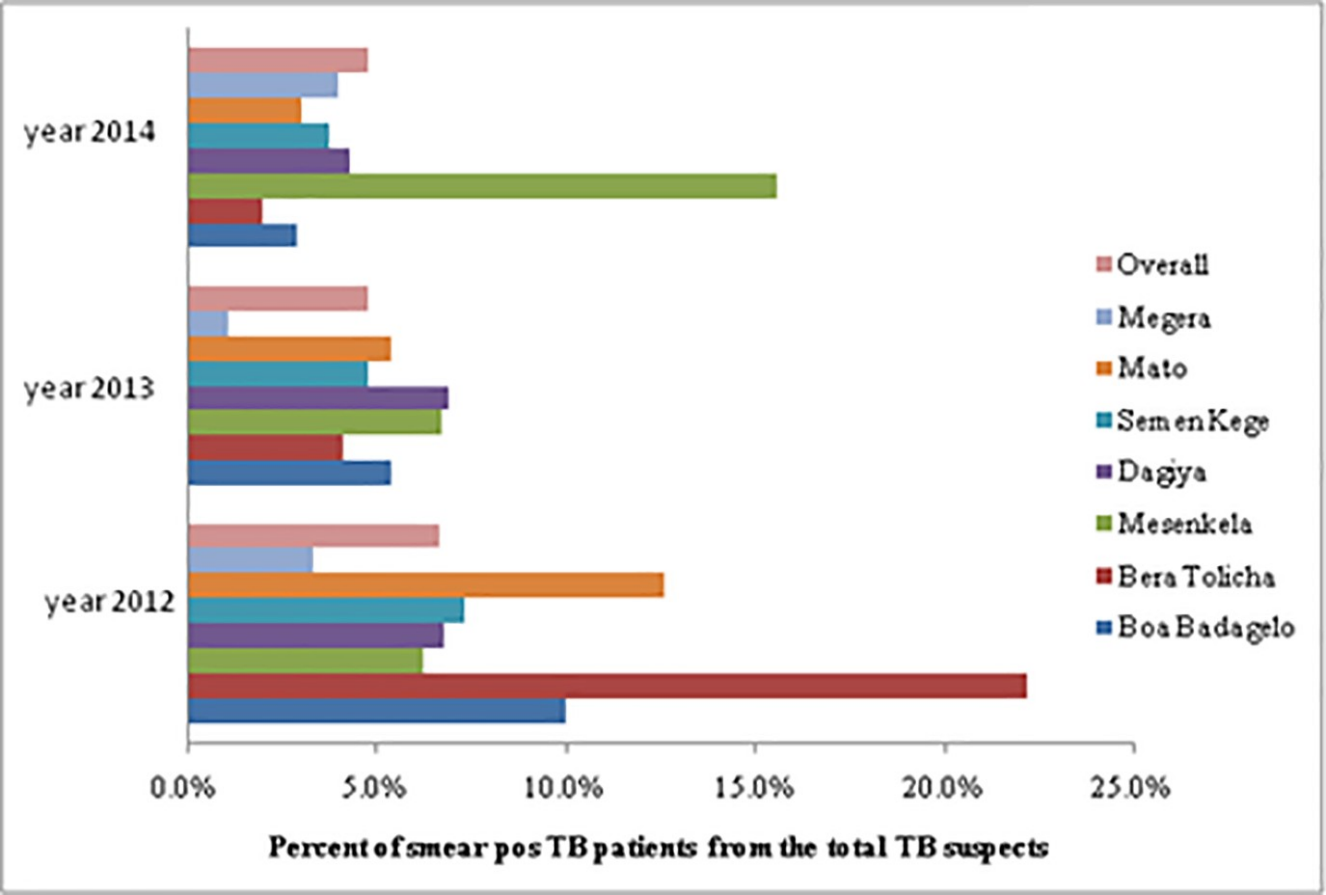
The proportion of smear-positive TB patients out of the total TB suspected patients over the three years (2012–2014) across health facilities of Dale district, Sidama Zone, Southern Ethiopia.

Overall, during the whole study period, only 5.3% of all TB suspected individuals were found to be smear-positive TB cases. The incidence rate of smear-positive TB to TB suspects in each study year consecutively was recorded to be 6.7%, 4.8%, and 4.8% (Fig 6). In most health centers, the number of TB patients to total TB suspects was less than 5%, Fig 6. The proportion of smear-positive TB patients was calculated by dividing the number of smear-positive cases by the total number of TB suspects.

### The case notification rate of smear-positive TB

**Fig 7:**
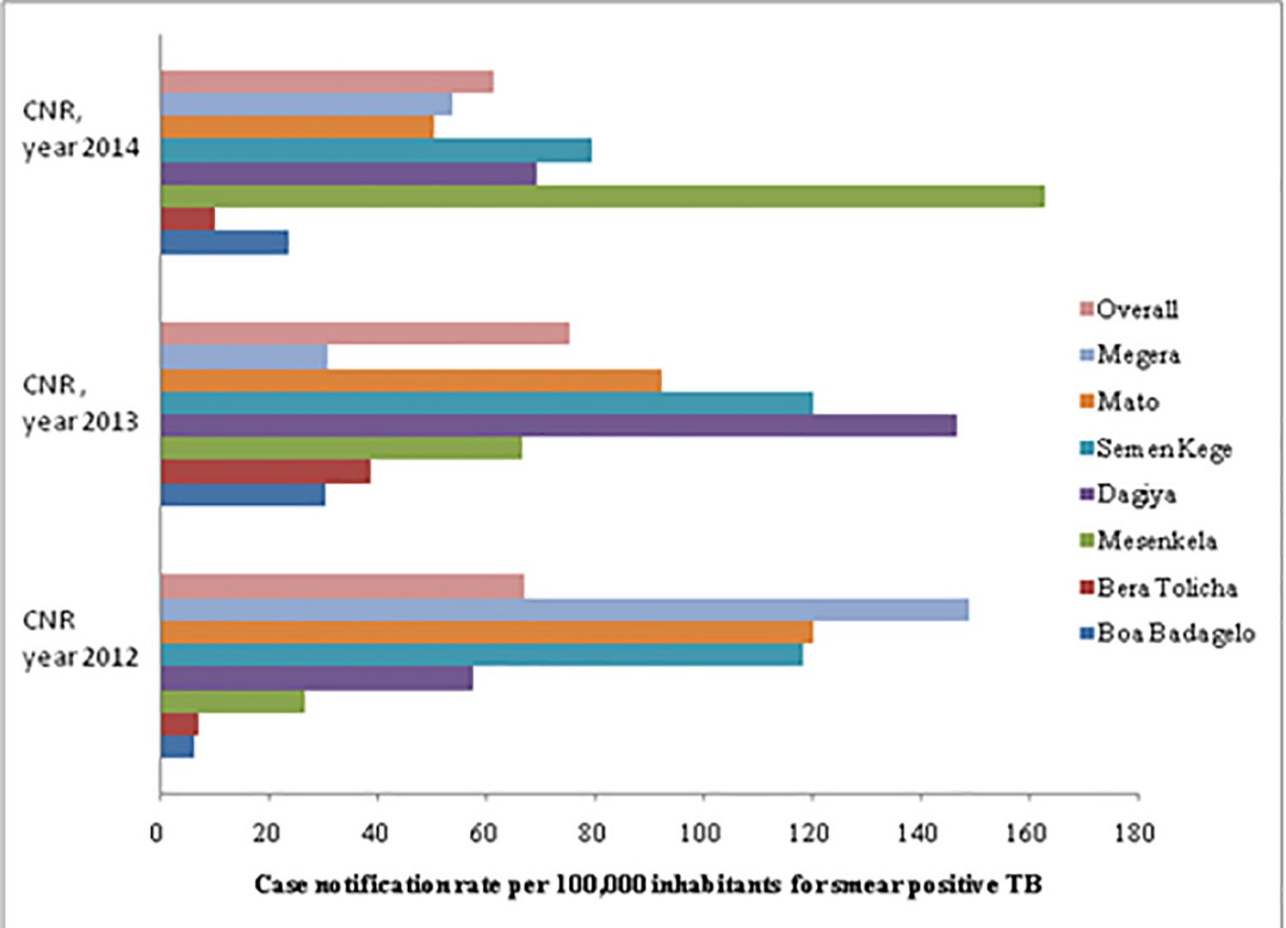
Notification rate for smear-positive TB over a period of time across the studied health facilities in Dale district, Sidama Zone, Southern Ethiopia.

There was some variation in the case notification rate of smear-positive TB by health centers, as was observed in the study. The highest notification rate, 163/100, 000 inhabitants, was reported from Mesenkela in 2014, Fig 7. The average notification rate for smear-positive TB cases in the year 2012, 2013, and 2014 was 67, 57, and 62 per 100, 000 inhabitants respectively. The notification rate was minimal for both extreme age groups, under five years’ old children and adults exceeding 50 (Fig 7 and Table 3). Only 6% of males and 4.8% of females had smear- positive TB. The odds of being diagnosed with smear-positive TB were 24% more in males than females though statistically not significant (adj OR = 1.24 (95% CI: 0.99–1.55, p = 0.08).

**Table 3:** The proportion of smear-positive and the odds of being diagnosed with smear-positive TB across different characteristics in Dale district, Sidama Zone, Southern Ethiopia.

Variable		% smear-positive (95%CI)	Crude OR	Adj OR	*p*-value
Age	< = 14 years	3.4 (1.8, 5.1)	Ref.	Ref.	
	15–24 years	8.2 (6.5, 9.9)	2.51 (1.45, 4.34)	2.50 (1.45, 4.33)	0.95
	25–34 years	5.5 (4.5, 6.5)	1.63 (0.95, 2.79)	1.74 (1.01, 2.97)	0.34
	35–50 years	4.4 (3.6, 5.1)	1.28 (0.76, 2.18)	1.33 (0.78, 2.26)	0.23
	> 50 years	6.5 (0.0, 15.2)	1.94 (0.43, 8.84)	2.21 (0.46, 9.70)	0.91
**Sex**	Female	4.8 (4.2, 5.4)	Ref.	Ref.	
	Male	6.0 (5.1, 6.8)	1.26 (1.03, 1.54)	1.24 (0.99, 1.55)	0.08
**Year**	2012	6.7 (5.6, 7.8)	Ref.	Ref.	
	2013	4.8 (4.1, 5.6)	0.71 (0.55, 0.90)	0.59 (0.45, 0.77)	0.02
	2014	4.8 (4.0, 5.6)	0.70 (0.54, 0.90)	0.66 (0.50, 0.87)	0.03

Adj = Adjusted, OR = Odds Ratio, Ref. = Reference group.

The majority (8.2%) of the smear-positive cases was found among the age group of 15 to 24 years. On the other hand, the number of smear-positive pulmonary tuberculosis cases was 6.7% in 2012 to 4.8% in both 2013 and 2014. The odds ratio between 2013 and 2014 was considerably lower when compared with 2012 (Table 3).

## Discussion

### Smear-positive TB notification rate

In this pioneer study, the sputum smear-positive pulmonary tuberculosis was 5.3% among all TB suspects. Smear positivity in each individual year (2012, 2013, and 2014) was appeared to show a decline from 6.7, 4.8, and 4.8% respectively. Despite under recommended implementation of TBIC measures in Dale district health facilities, such decline in case notification rate might either attributed to lack of diagnostic facilities, weak level of active surveillance systems, inadequate knowledge of patients with TB symptoms, less experienced health care workers for timely diagnosis of TB and, stigma at the community level or Dale district is on course of achieving reduction in TB cases, which actually needs further investigation [25]. The findings from this study are in agreement with similar studies done in West Gojam, Ethiopia, and South Africa [26, 27]. Unlike our finding, an increased case notification rate was reported from previous ten-year retrospective case notification rate of Sidama Zone, Southern Ethiopia [17]. In addition, the case notification rate of the present study is lower than the findings of the study done at Metehara sugar factory hospital, 14.2% [28]. This discrepancy could be attributed to differences in study design, several sample sizes, and differences in the duration of the study period.

Our study revealed that the odds of being diagnosed with smear-positive TB were 24% more in males than in females showing that males are more vulnerable than females to TB. The male predominance for frequent episode of TB coincides with data from other countries such as Southern Mexico and Bangladesh and might attributed to behavioural, occupational or immunological contributions to risk [29, 30]. In addition, Men are reported for frequent exposure to alcohol and tobacco, behaviours that have been attributed to develop disease after infection with TB [30, 31]. Furthermore, Studies obtained from developing countries, Vietnam and Zambia, have suggested that women have less access to health care and are less likely to undergo smear examination [32, 33]. However, active case finding via household visits help to reduce the concern of inequity of health care services among men and women.

Consistent with various studies throughout the world, tuberculosis is affecting everyoneregardless of age, but in our study most smear-positive TB cases were happened be in the age groups between 15 and 24. However, in developed countries, TB occurred among older age groups due to an aged population [34]. In developing countries, the high burden of TB is common among younger population [14]. The increasing in CNR among younger groups in our study was in agreement with data from the first national TB prevalence survey in Ethiopia [6, 14].

The average CNR of smear-positive TB at Dale district health centers was recorded to be 67, 57, and 62 per 100, 000 inhabitants in the three consecutive study years, namely, 2012, 2013, and 2014 respectively. This finding was noted to be similar with first Ethiopia national population-based tuberculosis survey, 63 /100,000 [6]. However, the CNR in this study is lower than studies conducted previously in Sidama Zone (CNR = 111) [17] and outreach program study in selected communities of Southern region of Ethiopia (CNR = 98) [22]. Moreover, the CNR of the population-based prevalence survey of tuberculosis in the Tigray region of Ethiopia was found to be 169 per 100,000 people [35], which was higher than the CNR of the current study. This discrepancy might be attributed to the variation in TB prevalence rate across different geographical settings. Similar pieces of evidence were also found in North Ethiopia and other African countries [36–40].

### Implementation of TB infection control plan

This study assessed the implementation of TBIC measures in 7 health facilities at Dale district using templates from the Ethiopian Federal Ministry of Health (FMOH, 2010), which was adopted from the WHO protocol and prepared to fit the Ethiopian health facility setting [21, 24, 41]. Despite the provision of necessary TB related services to the health facilities, the implementation of TBIC measures in these facilities was found to be under recommended standard. Health care workers (HCWs) as well as patients visiting these health facilities were not provided basic practical information which would enable them to render safe and effective TB care. Only 2/7 (28.6%) of the health care centers had TBIC plan measures and 5/7 (71.4%) of the facilities had assigned a focal person for the TBIC plan. Moreover, only 28.6% of the facilities monitored and evaluated TBIC activities. Facility-level managerial activities such as onsite surveillance of TB among HCWs were not conducted by any of the facilities under the study. This finding concurs with the study conducted in Vhembe district, Limpopo province of South Africa which identified the poor implementation of TBIC in association with TB transmission in health care facilities [42].

The result concerning the administrative measures varied among the facilities in terms of the total score and as a separate item. Only 42% of facilities were reported to identify patients with TB sitting next to other patients who visited the facilities. However, during the study, none of these facilities were found to practice triage and separation of infectious patients. The discrepancy between self-reported and observed was also demonstrated in other studies [19, 43, 44]. This inconsistency between reported and observed measures shows the limitation of self- reports.

Instrumental elements of administrative measures like package for prevention of HCWs from TB & HIV, waiting area for HIV and TB patients were not available in all facilities. Also, most of the facilities lack TB screening services for their staff members. Similar poor administrative measures have been reported in other settings like in Uganda [44].

The absence of separate well ventilated waiting areas for TB, HIV, and other patients would increase the nosocomial transmission of TB. It is important to appreciate TBIC recommended measures to reduce TB transmission amongst patients substantially [45]. All the facilities have been conducted periodic screening for all workers who have occupational exposure to TB which coincides with guidelines of FMOH, preventing the transmission of *M*. *tuberculosis* in health care settings [24]. TB infections among health workers were not be able to be predicted since any of the facilities had not reported TB infection among HCWs. All facilities conducted TB screening for HIV patients which is in contrast to similar studies done in Nigeria where there was no routine screening of HIV patients for TB infection [46]. In general, the finding of this study suggests the district and facilities managers should take corrective action in the administrative control measures which is considered as the first line of defense for the three level-hierarchy of TBIC plan [19, 43, 44].

The implementation of environmental control measures in the facilities was ranged between 16% and 83%. The findings from observations allowed us to notice a problem with building designs that needs structural changes in the studied facilities. As per the guidelines of FMOH [7], ventilation and proper airflow are considered to be important measures to prevent TB transmission in health facilities. However, some facilities lack cross ventilation and only 14% of the facilities had mechanical ventilation, which is considered to be a shortage; otherwise, mechanical ventilation is thought to increase airflow in the examination rooms and laboratories. Besides, there is a lack of UV germicidal to decontaminate working areas in the facilities. Similar studies from Nigeria have also reported a lack of such equipment [47]. These increase the possibility of the risk of TB transmission within the health facilities. All facilities reported to have adequate ventilation in the outdoor waiting and sputum collection area. On the contrary, studies done in South Africa found to have inadequate ventilation (in waiting areas and consultation rooms) [19], which indicates that the health facility setting in Dale district Southern part of Ethiopia is in a comparable or probably better footing than some of other countries with limited-resource settings.

Regarding the personal protective measures, most of the health care centers have a shortage of personal protective equipment. Only 14% of the facilities had N95 respirator for infectious TB patients. This precautionary measure is taken to be important as it could lead to higher risk of TB transmission. The more infected person is coughing profusely in the presence of other non-TB patients and HCWs, the higher risk of being exposed to TB infection. Furthermore, personal protective equipment (PPE) helps to protect the transmission of multi-drug resistant tuberculosis that occurred in the area [48].

Although the current study revealed the importance of facility-based data analysis at district level, it has also some limitations. The main drawback of this study is the absence of socio-economic and environmental data which is the inherent limitation of retrospective study. Hence, it could affect the result as it might have an association with the discrepancy in TB case notification rate. Retrospective nature of the health care action might also subject to recall bias and is taken as one of the constraints in this study. In addition, Due to Inadequate and less experienced stuff members in the health facilities, we may have missed many TB patients. Thus, our methodology probably underestimates some active TB cases. Furthermore, TBIC audit was done at a single particular time. Some facilities may have changed their infection control measures prior to the audit, which underestimate TBIC data in our study and could introduce the possibility of reverse causality. Future investigations should consider regular infection control audits and routine surveillance in health facilities.

## Conclusion

Regardless of some limitation in the study, the findings provide important information on the status of TB CNR and implementation of TBIC measures in the health facilities of Dale district, Sidama Zone, Southern Ethiopia. TB CNR was observed to be reduced in each consecutive year of the study period although the exact reason needs further investigation. Implementation of TBIC in Dale district health facilities was poor. TBIC practices implemented by each of the health facilities showed variation and only few health units had TBIC measures. Thus, adequate availability of TB diagnostic facilities and decentralization of TBIC measure leads to substantial gains in TB case notification and infection control practices, and is a key strategy in building more equitable and improved TB control efforts in Ethiopia. To this end, further research is recommended on the prevalence and incidence of tuberculosis to see the differences in the distribution of the disease and the performance of the on-going TB control programme strategy across the Dale district.

## Supporting information

S1 Appendix Questionnaire. (DOCX)
