# Supplementary material for: Combined assessment of tuberculosis case notification rate and infection control at health facilities of Dale districts, Sidama Zone, Southern Ethiopia
Source: PLoS One. 2021 Oct 12;16(10):e0242446. doi: 10.1371/journal.pone.0242446 (PMC8509858; doi:10.1371/journal.pone.0242446)
Supplement: S1 Appendix — (DOCX) [file pone.0242446.s001.docx]

**Check list to assess the implementation of TB infection control measures on Dale Woreda health facilities**

**Name of the Health Facility: ____________________________**

**Address: __________________**

**Name of Responsible Person for Infection Control in this Facility: ____________**

**Name of person administering the interview:______________________________________**

**Services provided in this facility**

* Please tick with √ Integrated TB-HIV Services  VCT/ART Services

 In-patient Services  TB Services

 GOPD

| SN | Question | Response |
| --- | --- | --- |
| **General information** | | |
| 1 | Type of facility? |  |
| 2 | How many full time staff work at this Facility? |  |
| 3 | In the each year, how many staff members were  diagnosed with TB? |  |
| 4 | Out-patient turn over/year |  |
| 5 | TB patient /year |  |
| 6 | TB diagnosis (on site or off-site) |  |

| SN | Questions | Response | |
| --- | --- | --- | --- |
| 1. **Administrative** | | | |
|  |  | **YES** | **NO** |
| 1 | Is there an IC team or responsible person in place? |  |  |
| 2 | Is there a Facility IC plan in place? |  |  |
| 3 | Has a facility risk assessment been conducted in the facility? |  |  |
| 4 | Is ‘’on-site’’ surveillance on TB disease among staff being conducted (including monitoring and evaluation of IC) |  |  |
| 5 | Is health education on IC ensured for HCWs? |  |  |
| 6 | Is health education on IC ensured for patients, and visitors? |  |  |
| 7 | Does the facility participate in operational research (OR)? |  |  |
| 8 | Which of the following recommended controls are practiced? |  |  |
|  | • Triage; |  |  |
|  | • Separation; |  |  |
|  | • Cough etiquette; |  |  |
|  | • Expedient service delivery (prompt services for ‘’coughers’’) |  |  |
| 9 | Package of prevention for HCWs, including HIV prevention, ART and IPT for HIV-positive staff |  |  |
| 10 | Do you screen HIV patients for active TB? |  |  |
| 11 | Is there a separate sites for HIV care and treatment in the facility? |  |  |
| 12 | Are posters on cough hygiene prominently  Displayed? |  |  |
| 13 | Is there a symptom checklist in place to screen patients for TB? |  |  |
| 14 | Are staff members screened for TB? |  |  |
| 15 | Are staff members offered confidential HIV counseling and testing? |  |  |
| 16 | Ensuring adherence and completion of treatment of TB cases |  |  |
| 1. **ENVIRONMENTAL** | | | |
|  |  | **YES** | **NO** |
| 1 | Natural ventilation in place, especially in waiting areas, examination room, and sputum collection room |  |  |
| 2 | Mechanical ventilation in place, especially in waiting areas, examination room and sputum collection room |  |  |
| 3 | Is there a Cross-ventilation of windows and doors for air movement especially in waiting areas, examination room and sputum collection room |  |  |
| 4 | Outdoor waiting areas or open space |  |  |
| 5 | Ultraviolet-Germicidal Irradiation (UVGI) |  |  |
| 6 | Is there electricity at this facility? |  |  |
| 1. **Respiratory PROTECTION** | | | |
|  |  | **YES** | **NO** |
| 1 | Respirators available for staff |  |  |
| 2 | Fit testing and/or fit check for respirators |  |  |
| 3 | Are surgical available for coughing patients? |  |  |
| 4 | Are N95 masks available for coughing patients? |  |  |
